# Utilization of Deworming Drugs and Its Individual and Community Level Predictors among Pregnant Married Women in Cameroon: A Multilevel Modeling

**DOI:** 10.1155/2021/6645336

**Published:** 2021-05-12

**Authors:** Betregiorgis Zegeye, Bright Opoku Ahinkorah, Edward Kwabena Ameyaw, Abdul-Aziz Seidu, Sanni Yaya

**Affiliations:** ^1^Shewarobit Field Office, HaSET Maternal and Child Health Research Program, Addis Ababa, Ethiopia; ^2^School of Public Health, Faculty of Health, University of Technology Sydney, Australia; ^3^Department of Population and Health, University of Cape Coast, Cape Coast, Ghana; ^4^College of Public Health, Medical and Veterinary Sciences, James Cook University, Townsville, Queensland, Australia; ^5^University of Parakou, Faculty of Medicine, Parakou, Benin

## Abstract

**Background:**

Although deworming pregnant women is one of the strategies to reduce parasites (roundworms and hookworms) causing anemia and related perinatal and maternal complications, utilization of deworming medication among pregnant women in Cameroon is suboptimal. Comprehensive assessment of individual, household (including women's autonomy), and community-level factors associated with utilization of deworming medication has not been done so far. Therefore, we investigated the individual/household and community-level factors associated with deworming among pregnant married women in Cameroon.

**Methods:**

Our study was limited to pregnant women because they have a greater risk due to increased chances of anemia. We used data from the 2018/19 Cameroon Demographic and Health Survey. Analysis on 5,013 pregnant married women was carried out using multilevel logistic regression. Odds ratios with a 95% confidence interval (CI) were reported.

**Results:**

Our findings showed that about 29.8% of pregnant married women received deworming medications. The individual/household level predictors of deworming medications utilization identified in this study were women's educational level, wealth quintile, and skilled antenatal care. Distance to health facility and region were identified as community-level predictors of deworming medications utilization. Higher odds of receiving deworming medication occurred among educated and wealthier pregnant married women as well as among pregnant married women who had skilled antenatal care or lived in the south region, whereas lower odds were observed among pregnant married women living in the north region.

**Conclusion:**

Access to education and economic empowerment of pregnant married women in remote areas and the north region should be the primary focus of the Cameroon government to enhance deworming coverage in the country.

## 1. Background

Soil-transmitted helminths (STH) are one of the major public health problems as it predominantly affects people in low- and middle-income countries [1–3]. Globally, nearly 1.3 billion people are affected by STH [1], and in 2010, STH caused 4.98 million disability-adjusted life years [2]. Evidence in 102 STH-endemic countries in 2015 [4] revealed that STH infected more than 2 billion people who in turn lose an estimated 39 million disability-adjusted life years [4]. The problems of STH are highly prevalent in South East Asia and sub-Saharan African countries, especially in the tropical and sub-tropical parts of sub-Saharan Africa (SSA) [5]. In Africa, about ten million pregnant women suffer from anemia due to schistosomiasis and about 7 million pregnant women in SSA are infected with hookworms [6]. STH are common diseases and distinctive among communities with inadequate infrastructure, hygiene, or sanitation facilities and among communities with poor awareness of the benefits of proper face disposal [7].

There are three high-risk groups; women of reproductive age, preschool age, and school-age children in which STH become more severe and may even lead to death once infected [4]. Within women of reproductive age, pregnant women have a higher risk compared to nonpregnant women. Due to the intensity of anemia in pregnancy, they become more worsened if they acquire additional infestation of helminths, especially by hookworms as they are less resistant to infection [8, 9]. Anemia has several effects on pregnant women including increased susceptibility to infection, stillbirth/miscarriage [10, 11], and poor feto-maternal outcomes such as preterm birth, low birth weight, and infant or child mortality [11–13]. In pregnancy, infections are the major causes of anemia, which can be prevented by taking deworming medication, sleeping in bed net and taking intermittent preventive malaria treatment [14]. In addition to the individual level effect, anemia has huge health and adverse economic effects for communities and nations [14].

Communities and national health and economic adverse effects of anemia are greatly seen; due to the societal consequences of increased maternal mortality and resultant restraints on productivity, the costs incurred by the public and private sectors in the therapeutics measures for the prevalent level of anemia and the long-term projected negative consequence of impaired mental development on the human capital formation [15]. However, the degree of communities and national health and economic adverse effects depend on the number of individuals affected by anemia, severity of anemia, and duration and consequence of the condition [15]. Moreover, among children, significant growth retardation and delayed cognitive development are common and associated with *Trichuris trichiura* and *Ascaris lumbricoides* [16, 17].

As a public health intervention, WHO recommended deworming or preventive chemotherapy (PC) for pregnant women after first trimester, using a single dose of mebendazole (500 mg) or albendazole (400 mg) in areas where there is 40% or higher anemia prevalence among pregnant women and the prevalence of *T. Trichiura* and *hookworm* are 20% and above [18]. As documented in several prior studies, prevention chemotherapy or deworming of pregnant women and children significantly enhance hemoglobin level and nutritional status [19–21], and as a result, their wellbeing can be improved whilst huge reduction of morbidity and mortality can be achieved [22]. Mothers who are dewormed during pregnancy tend to have an improved birth weight of their babies [23, 24]. Moreover, a reduction of the prevalence of very low birth weight (birth weight less than 1500 grams) is seen among women who take deworming medicine during pregnancy [25].

Cameroon is one of the STH endemic countries with moderate STH infestation where no substantial progress has been observed in its reduction [26]. In Cameroon, nearly half of pregnant women (49.3%) were anemic as of 2016 [27]; however, there is low coverage of deworming medication [14]. For instance, according to the 2011 Cameroon Demographic and Health Survey (CDHS), less than 40% of pregnant women received deworming medication [14]. Evidence shows that individual and communities' socioeconomic status and geographic-related factors affect the uptake of deworming medication [28]. Besides, married women are less likely to use maternal health services including uptake of deworming medication due to low autonomy or participation in decision-making within the household [29, 30] and gender inequality or male dominance [29–32]. Regarding socioeconomic factors, for instance, noneducated pregnant women are less likely to take deworming medication due to poor awareness and utilization of maternal health services, including antenatal care (ANC) that comprises supplementation of deworming medication [33–35]. Women in the poorest household are more likely to be challenged by the problem of affordability of transportation to health facility [36]. Moreover, women living in distant and rural areas and regions with underdeveloped infrastructures such as road are less likely to take deworming medication from a health facility or drug stores [37].

To the best of our knowledge, a comprehensive assessment of predictors of deworming medication among pregnant married women has not been done in Cameroon. Hence, the purpose of this study was to determine the coverage and individual/household and community-level predictors of utilization of deworming among married women during pregnancy in Cameroon. The findings from this study could help to enhance national deworming medication coverage by intervening on current individual/household and community level predictors, which can potentially reduce helminthic-related anemia among pregnant married women in the country.

## 2. Methods

### 2.1. Data Sources and Sampling Procedure

The data used for the analysis in this study were extracted from the 2018/19 Cameroon Demographic and Health Survey (CDHS), which is carried out by the Cameroon National Institute of Statistics (CNIS) in collaboration with the Ministry of Health (MOH) with financial and technical support from the United States Agency for International Development (USAID) and ICF International [38]. The CDHS collects data to produce evidence for monitoring vital population and several health indicators including utilization of deworming medications [38].

In the CDHS, the two-stage stratified cluster sampling technique was applied. In the first stage, primary sampling units (PSUs) or enumeration areas (EAs) were selected from the sampling frame, which was prepared from the recent population census using probability proportional to size [38]. In the second stage, a fixed number of households [25–30] were selected from the selected EAs using a systematic sampling technique [38]. A total of 14,677 women aged 15-49 and 6,978 men aged 15-64 were interviewed from 11,710 households [38]. Detailed descriptions of the methodology used in the survey are explained in the final report of the 2018/19 CDHS [38]. For this study, we used the Individual Recode (IR) file and limited the analysis to a sample of 5,013 pregnant married women. We used IR file because datasets for measuring women's health indicators such as the utilization of deworming medication are found in that file [39]. In addition, we limited the study to married women because female empowerment factors such as decision-making power were confined to only married women [39–41].

### 2.2. Study Variables

#### 2.2.1. Outcome Variable

Utilization of deworming medication was the outcome variable for this study. The WHO recommends that pregnant women take a single dose of mebendazole (500 mg) or albendazole (400 mg) after the second trimester [18]. The DHS asked pregnant women who took deworming medication with a birth in the last five years [18, 39, 42]. We categorized and coded responses to binary as “yes” if they took and “no” if they did not take the deworming medication.

#### 2.2.2. Explanatory Variables

We incorporated several individual/household and community level explanatory variables based on available evidence on the uptake of deworming medication among pregnant women [19–25, 28, 43]. We incorporated the following individual/household level predictors and coded them as follows: maternal educational level (no education, primary, secondary, higher), husband's educational level (no education, primary, secondary, higher), women's occupation (not working, clerical, sales, agricultural self-employed, services, skilled manual, unskilled manual), husband's occupation (not working, professional or technical or managerial, clerical, sales, agricultural self-employed, service, skilled manual, unskilled manual) religion (Catholic, Protestant, Other Christians, Muslim, Other), sex of household head (male, female), and skilled antenatal care (ANC) (no, yes). The wealth index was coded as poorest, poorer, middle, richer, and richest. In DHS, for measuring households' economic status, the wealth index is usually computed using durable goods, household characteristics, and basic services following the methodology explained elsewhere [44], and we followed the same procedure. Regarding media exposure (yes, no), we coded yes if the women read newspaper, listened radio, or watched television for at least less than once a week, and no for otherwise. Women's decision-making power was coded as yes versus no. If the women decided, either alone or together with their husband on all three of decision-making parameters; their own health, to purchase large household expenses, to visit families or relatives, the women considered as having decision-making power. However, if the woman did not decide, either alone or together with her husband, on at least one of the three abovementioned decision-making parameters, the woman was considered as having no decision-making power.

The community-level factors included in this study were as follows: distance to health facility (big problem, not a big problem) place of residence (urban, rural), and region (Adamawa, Centre [without Yaoun], Douala, East, Far-North, Littoral [without Dou], North, North-West, West, South, South-West, Yaounde). Others were community literacy level (low, medium, high) and community socioeconomic status (low, moderate, high). In this study, a big problem indicates that the distance from women's home to health facility (could it be a health center or hospital) to get medical help for herself was problematic. If the women responded as the distance was a big problem, we coded as 1 if the women reported as not a big problem and coded as 0 if the women were reported as a big problem. The socioeconomic status variable was an aggregation from occupation, wealth, and education of research participants who resided in a given community. We further applied principal component analysis to estimate women who were unemployed, uneducated, and poor. A standardized score was derived with a mean score (0) and standard deviation [1]. The scores were then segregated into tertile 1 (least disadvantaged), tertile 2, and tertile 3 (most disadvantaged), where the least score (tertile 1) denoted greater socioeconomic status and the highest score (tertile 3) denoting lower socioeconomic status. Community literacy level was derived from women who could read and write (or not read and write) at all.

### 2.3. Statistical Analyses

First, descriptive analysis including frequency distribution of respondents, utilization of deworming medication, and utilization across explanatory variables was conducted. Then, a chi-square test of independence was carried out to select variables that had a significant association with utilization of deworming medications at *P* value 0.05 cut point. Subsequently, a multicollinearity test was done using variance inflation factor (VIF) for all statistically significant variables at the chi-square test, and we found no evidence of high collinearity among the explanatory variables (Mean VIF = 1.71, Min VIF = 1.03, Max VIF = 3.51). Based on available evidence, a mean VIF less than 10 is acceptable [45, 46].

Finally, four different models were constructed using the multilevel logistic regression (MLLR) technique to assess whether or not the individual/household and community level predictors had significant associations with the outcome variable (utilization of deworming medication). The first model was a null model, which had no explanatory variables, and it displayed variance in the coverage of deworming medication, attributed to PSU. The second model called model I incorporated only the individual/household level predictors and the third model (Model II) included community-level predictors only. The final model, (Model III), comprised both the individual/household and community level predictors.

All four MLLR models included fixed and random effects [47–49]. The fixed effects indicated the association between the explanatory variables and the outcome variable and the random effects signified measure of variation in the outcome variable based on PSU, which is measured by intracluster correlation (ICC) [50]. Finally, the model fitness, or how the different models were fitted with the data, was examined using Akaike's Information Criterion (AIC) [51]. We used the “mlogit” command to run the MLLR models. Weighting was done to take into account the complex nature of DHS data, while the “svyset” command was used for adjusting for disproportionate sampling and nonresponse. The analysis was conducted using the Stata version-14 software (Stata Corp, College Station, Texas, USA).

### 2.4. Ethical Considerations

We used publicly available DHS data from MEASURE DHS for analysis of this study. Since the institution commissioned, funded, and managed the survey, further ethical clearance is not required. ICF international ensured that the protocol of the survey was compliant with the U.S. department of health and human service regulations to protect human subjects.

## 3. Results

### 3.1. Sociodemographic Characteristics of Respondents

In this study, a total of 5,013 pregnant married women participated. Of them, 52.5% were rural residents. Nearly 40% of the respondents had attended secondary school, while 31.1% and 29.6% had attended primary school and not had formal education, respectively. More than 89% of the respondents were followers of Catholic (34.9%), Muslim (30.3%), and Protestant (23.9%) faiths, respectively. About 85.9% of the married women had skilled ANC. Regarding women's decision-making capacity, about 47.7% of women decided in all of the three decision-making parameters (their own health, large household expenses, and visiting family/relative) either alone or together with their husband (Table 1).

### 3.2. Utilization Coverage of Deworming Drugs across Explanatory Variables

Table 1 shows variations in the utilization of deworming drugs among married women across various subpopulations. For instance, the utilization among pregnant married women who had attended secondary and higher school was 41.1% and 40.2%, respectively, but it was 16.2% among pregnant married women who had no formal education. Utilization also varied from 16.3% among married pregnant women in the poorest subgroup to 40.6% and 42.6% among pregnant married women in the richer and richest subgroups, respectively. Approximately 39.2% of pregnant married women who had media exposure (newspaper, radio, or television) took deworming drugs, whereas nearly 22.4% of pregnant married women who had no media exposure took the drugs. About 54.1% of women living in the south region took the deworming drugs; however, only 9.1%, 23.3%, and 24.8% of pregnant women in North, Adamawa, and Far-North regions, respectively, took the deworming drugs (Table 1).

### 3.3. Measure of Association (Fixed Effects) Results

#### 3.3.1. Individual/Household Level Predictors

The study shows that women's educational status, economic status, and skilled ANC were significant individual/household level predictors for utilization of deworming drugs among pregnant married women in Cameroon. We found that the likelihood of taking deworming drugs among pregnant married women who had attended secondary school (aOR = .38, 95% CI; 1.04-1.83) was higher as compared to pregnant married women who had no formal education. Moreover, the study shows that the likelihood of taking deworming medication among pregnant married women in the richest (aOR = 1.90, 95% CI; 1.25-2.88), richer (aOR = 1.69, 95% CI; 1.17-2.44), and middle (aOR = 1.39, 95% CI; 1.01-1.90) categories were higher, respectively, as compared to pregnant married women in the poorest categories. We found higher odds of the utilization of deworming medication among pregnant married women who had skilled ANC (aOR = 18.54, 95% CI; 11.05-31.10) as compared to pregnant married women who had no skilled ANC (Table 2).

#### 3.3.2. Community Level Predictors

This study shows higher odds of the utilization of deworming medication among pregnant married women who reported distance to health facility was not a big problem (aOR = 1.19, 95% CI; 1.02-1.39) as compared to women who reported distance to health facility was a big problem. Significant regional variations in the utilization of deworming medication among pregnant married women were observed. In fact, we found higher odds of the utilization of deworming medication among pregnant married women living in the South region (aOR = 2.09, 95% CI; 1.27-3.45) as compared to pregnant married women living in the Adamawa region. However, the likelihood of taking deworming medication among pregnant married women living in the North region was lower (aOR = 0.27, 95% CI; 0.17-0.43) as compared to pregnant married women living in the Adamawa region (Table 2).

### 3.4. Measures of Variations (Random Effects) Results

The results of the Model 0 of Table 2 show that coverage of deworming medication varies significantly across the clusters (*σ*^2^ = 1.45, 95% CI; 1.17-1.80). Model 0 revealed that 30% of coverage of deworming medication was related to the between-cluster variations (ICC = 0.30). The between-cluster difference decreased from 30% in the Null model (Model 0) to 23% in the model that had only the individual/household level variables (Model I). The between-cluster difference decreased from 23% in the Model I to 21% in the community level only model (Model II) and again to 18% in the complete model (Model III) that included both individual/household and community level factors. This specifies that the differences in the coverage of deworming medication can be clarified by the variances across the clusters (Table 2).

## 4. Discussion

In this study, the individual/household and community-level predictors of deworming medication among pregnant married women during pregnancy were thoroughly investigated using data from the 2018/19 CDHS. The findings show that the utilization of deworming medication in Cameroon was 29.8%. We found women's education, household economic status, and skilled ANC to be the individual/household level predictors of utilization of deworming medication, whereas the distance to health facility and region were the community level predictors.

More specifically, we found that the likelihood of taking deworming medication was higher among pregnant married women who had attended secondary school compared to pregnant married women who had no formal education. Several studies confirm that there is a significant association between maternal educational status and maternal health service utilization such as ANC [33, 34] and uptake of deworming medication [28]. The possible reason for better uptake of maternal health service among educated women might be due to higher health knowledge among educated women as compared to noneducated [35]. Not only that educational attainment has also been considered as an important social determinant of maternal, newborn, and child health [52]. Education encourages women to have autonomy, self-confidence, and health information and make an informed decision about their health [53, 54].

We found higher odds of taking deworming medication among pregnant married women in higher economic status compared to the poorest pregnant women. A previous study in Ghana reported similar findings [28]. Having a better socioeconomic situation as a woman facilitates not only access or affordability of transport costs but also helpful in buying the medication whenever the health facilities have supply problem or not available in dispensary or drug store [36]. Evidence shows that the early beginning of ANC service [55] and higher uptake of ANC service are seen among pregnant married women in better socioeconomic status as compared to women in lower economic status [33, 34, 55, 56].

The burdens of helminthic infection are prevalent among countries and communities with low socioeconomic status [57]. This evidence is supported by WHO's finding that showed the burden of maternal anemia is highly prevalent in South-East Asia and Africa, where higher parasitic infections are also prevalent [58]. It is known that anemia has negative consequences for perinatal and neonatal as well as maternal health [42]. Evidence shows that complicated and worse outcomes of pregnancy among disadvantaged women such as poorest/poor, uneducated, and rural residents are very common in low- and middle-income countries [59]. Hence, supporting women in such locations is required to lessen their suffering from the problem and poverty as well as to achieve equitable health service [57].

Skilled ANC was found to have a positive influence on the uptake of deworming medication. The odds of taking deworming medication among pregnant married women who had skilled ANC were higher compared to pregnant married women who had no skilled ANC. The evident justification for a higher uptake of deworming medication among pregnant married women with skilled ANC could be due to the fact that WHO recommended to routinely provide this medication for all women after the first trimester especially among the community with a high prevalence of STH and anemia among women for positive pregnancy outcome [42]. Therefore, the higher association between skilled ANC and uptake of deworming medication indicating that strengthening ANC service could substantially increase the coverage of deworming medication in the country [43].

This study shows that the likelihood of taking deworming medication among pregnant married women who reported distance to a health facility is not a big problem was higher compared to pregnant married women who reported the distance to a health facility is a big problem. In Cameroon, distances to health facilities are big problems for about 40% of the women [38]. According to 2018 CDHS, 72% of women aged 15-49 encountered problems in accessing health care services for at least one of the four reasons: getting permission, money, going alone, or distance to a health facility [38]. Scholars have documented that low economic and physical distance to health facilities, where poor individual mostly living farther from the health facility than the rich individual [60, 61]. The capacities to afford health service significantly affect the utilization of health service [62]. Hence, even if the health service is required for them, because of the influence of long distance to health facilities, poor uptake of maternal and child health services is seen in many studies [37, 63, 64].

We found significant variation in the odds of having deworming medication among pregnant married women across regions where they lived. More specifically, compared to pregnant married women living in the Adamawa region, we found that the likelihood of taking deworming medication among pregnant married women who were living in the South region was higher, while lower odds are reported among women in the North region. Evidence in Cameroon shows that maternal and child health services are varied across regions, where many of the maternal services including ANC and skilled delivery services remains very low, and high child mortality is observed [65]. For instance, according to the 2014 Cameroon MICS report, under-five mortality in the North region was 173 per 1000 live birth, which was higher as compared to the national average of 103 deaths per 100 live births and 52 deaths per 100 live births in both Yaounde and Douala regions [65, 66]. Similarly, based on the skilled delivery attendance in the North region was 35% while 99% in the Douala region [65].

### 4.1. Strengths and Limitations of Study

The major strength of our study lies in the use of data from the most recent nationally representative demographic and health survey. Again, producing up-to-date evidence about coverage and individual/household and community level predictors could reveal the country's gaps from the ideal target and recognizing the current predictors. Findings from our study can help to evaluate and revise plans regarding the provision of deworming medication among pregnant married women in Cameroon. The robust nature of methodology used in DHS such as multistage sampling with a high response rate can increase the generalizability of results to all pregnant women in Cameroon. Using advanced statistical models that consider individual/household and community level predictors also increase the quality of the paper. However, the study has the following limitations. First, variables not found in the dataset such as drug supply and other variables like perception and attitude were not covered. Second, the cross-sectional nature of the data does not allow for a cause-effect relationship. Finally, since the data are self-reported, the finding might be affected by recall bias.

## 5. Conclusions

The findings show women's educational level, household economic status, and skilled ANC are significant individual/household level predictors, whereas the distance to health facility and region was the community-level predictors of deworming medication utilization in Cameroon. Therefore, women empowerment through education and economy as well as improving the economic capacity of poor households could help in enhancing uptake of deworming medication. Moreover, giving attention to pregnant women living in far distances from health facilities and the north region residents can help in achieving equity-based maternal health service utilization such as the use of deworming medication in the country.

## Figures and Tables

**Table 1:** Coverage of deworming medication utilization among married pregnant married in Cameron: evidence from 2018/19 CDHS.

Variables	Frequency	Unweighted percent	Weighted percent	Deworming		Chi-square, *P* value
				No, freq. (perc.)	Yes, freq. (perc.)	
Maternal educational level						*χ* ^2^ = 226.51, *P* < 0.001
No education	2,070	25.68	29.56	1,079 (83.84)	208 (16.16)	
Primary school	2,721	33.76	31.1	1,102 (66.99)	543 (33.01)	
Secondary school	2,868	35.58	33.99	1,075 (58.90)	750 (41.10)	
Higher	401	4.98	5.35	153 (59.77)	103 (40.23)	
Husband educational level						*χ* ^2^ = 133.17, *P* < 0.001
No education	1,876	23.28	26.17	943 (80.32)	231 (19.68)	
Primary school	2,590	32.13	31.31	1,094 (68.81)	496 (31.19)	
Secondary school	2,899	35.97	33.67	1,128 (61.20)	715 (38.80)	
Higher	695	8.62	8.85	244 (60.10)	162 (39.90)	
Women's occupation						*χ* ^2^ = 56.04, *P* < 0.001
Not working	2,153	26.71	25.54	1,011 (71.05)	412 (28.95)	
Clerical	116	1.44	1.42	37 (61.67)	23 (38.33)	
Sales	1,638	20.32	20.05	570 (61.69)	354 (38.31)	
Agricultural self-employed	2,865	35.55	36.45	1,288 (72.00)	501 (28.00)	
Services	791	9.81	9.8	279 (59.11)	193 (40.89)	
Skilled manual	385	4.78	4.99	181 (64.41)	100 (35.59)	
Unskilled manual	112	1.39	1.75	43 (67.19)	21 (32.81)	
Husband occupation			.			*χ* ^2^ = 93.09, *P* < 0.001
Not working	347	4.31	3.73	58 (59.18)	40 (40.82)	
Professional/technical/managerial	318	3.95	3.77	183 (61.41)	115 (38.59)	
Clerical	147	1.82	1.64	912 (66.09)	468 (33.91)	
Sales	1,324	16.43	15.95	1,534 (74.90)	514 (25.10)	
Agricultural self employed	3,229	40.06	41.1	613 (59.28)	421 (40.72)	
Service	913	11.33	11.02	109 (70.32)	46 (29.68)	
Skilled manual	1,512	18.76	19.18	58 (59.18)	40 (40.82)	
Unskilled manual	270	3.35	3.61	183 (61.41)	115 (38.59)	
Economic status			.			*χ* ^2^ = 191.06, *P* < 0.001
Poorest	1,415	17.56	21.25	831 (83.69)	162 (16.31)	
Poorer	1,732	21.49	20.53	810 (70.74)	335 (29.26)	
Middle	1,893	23.49	19.91	747 (66.11)	383 (33.89)	
Richer	1,593	19.76	19.23	577 (59.36)	395 (40.64)	
Richest	1,427	17.70	19.08	444 (57.44)	329 (42.56)	
Religion						*χ* ^2^ = 86.69, *P* < 0.001
Catholic	2,805	34.80	34.85	1,084 (63.88)	613 (36.12)	
Protestant	2,138	26.53	23.89	820 (63.13)	479 (36.87)	
Other Christians	579	7.18	6.38	254 (66.32)	129 (33.68)	
Muslim	2,227	27.63	30.26	1,101 (75.77)	352 (24.23)	
Other	311	3.86	4.62	150 (82.87)	31 (17.13)	
Media exposure						*χ* ^2^ = 154.28, *P* < 0.001
No	3,374	41.86	44.16	1,681 (77.36)	492 (22.64)	
Yes	4,686	58.14	55.84	1,728 (60.85)	1,112 (39.15)	
Decision-making						*χ* ^2^ = 44.11, *P* < 0.001
No	3,961	49.14	52.31	1,938 (72.07)	751 (27.93)	
Yes	4,099	50.86	47.69	1,471 (63.30)	853 (36.70)	
Sex of HH head						*χ* ^2^ = 12.45, *P* < 0.001
Male	6,925	85.92	86.79	2,995 (68.91)	1,351 (31.09)	
Female	1,135	14.08	13.21	414 (62.07)	253 (37.93)	
Skilled ANC						*χ* ^2^ = 326.97, *P* < 0.001
No	695	13.86	14.02			
Yes	4,318	86.14	85.98			
Distance to HF						*χ* ^2^ = 56.49, *P* < 0.001
Big problem	3,211	43.03	42.41	1,622 (73.59)	582 (26.41)	
Not a big problem	4,252	56.97	57.59	1,787 (63.62)	1,022 (36.38)	
Place of residence						*χ* ^2^ = 54.46, *P* < 0.001
Urban	3,774	46.82	47.46	1,394 (62.57)	834 (37.43)	
Rural	4,286	53.18	52.54	2,015 (72.35)	770 (27.65)	
Region						*χ* ^2^ = 351.94, *P* < 0.001
Adamawa	690	8.56	5.29	330 (76.74)	100 (23.26)	
Centre (without Yaoun)	826	10.25	9.53	338 (64.88)	183 (35.12)	
Douala	555	6.89	10.11	176 (59.26)	121 (40.74)	
East	718	8.91	6.26	301 (63.91)	170 (36.09)	
Far-North	1,051	13.04	18.26	555 (75.20)	183 (24.80)	
Littoral (without Dou)	495	6.14	3.45	191 (67.97)	90 (32.03)	
North	1,017	12.62	15.64	621 (90.92)	62 (9.08)	
North-West	383	4.75	5.75	132 (53.23)	116 (46.77)	
West	788	9.78	10.68	310 (63.66)	177 (36.34)	
South	778	9.65	4.69	196 (45.90)	231 (54.10)	
South-West	164	2.03	1.58	49 (55.68)	39 (44.32)	
Yaounde	595	7.38	8.76	210 (61.40)	132 (38.60)	
Community literacy level			.			*χ* ^2^ = 197.26, *P* < 0.001
Low	3,296	40.89	44.74	1,724 (78.12)	483 (21.88)	
Medium	2,532	31.41	27.2	944 (62.89)	557 (37.11)	
High	2,232	27.69	28.06	741 (56.78)	564 (43.22)	
Community socioeconomic level						*χ* ^2^ = 113.05, *P* < 0.001
Low	3,973	49.29	50.03	1,942 (74.58)	662 (25.42)	
Moderate	1,766	21.91	19.01	686 (63.34)	397 (36.66)	
High	2,321	28.80	30.96	781 (58.90)	545 (41.10)	

**Table 2:** Multilevel multivariable results for individual/household and community-level predictors of utilization of deworming medication among pregnant women: evidence from 2018/19 CDHS.

Variables	Model 0	Model I AOR [95% CI]	Model II AOR [95% CI]	Model III AOR [95% CI]
Women's educational status				
No formal education (ref)				
Primary school		1.38 (1.08-1.77)^∗^		1.27 (0.99-1.64)
Secondary school		1.53 (1.16-2.02)^∗∗^		1.38 (1.04-1.83)^∗^
Higher		1.43 (0.93-2.22)		1.28 (0.82-1.98)
Husband educational status				
No formal education (ref)				
Primary school		0.91 (0.72-1.15)		0.85 (0.67-1.07)
Secondary school		0.92 (0.72-1.17)		0.86 (0.67-1.09)
Higher		0.87 (0.60-1.25)		0.83 (0.58-1.19)
Women occupation				
Not working (ref)				
Clerical		0.92 (0.51-1.69)		0.88 (0.48-1.60)
Sales		1.18 (0.96-1.45)		1.19 (0.97-1.46)
Agricultural self-employed		1.22 (0.98-1.52)		1.23 (0.98-1.54)
Services		1.08 (0.83-1.39)		1.07 (0.83-1.38)
Skilled manual		0.92 (0.68-1.24)		0.93 (0.69-1.26)
Unskilled manual		1.14 (0.63-2.07)		1.10 (0.61-1.98)
Husband occupation				
Not working (ref)				
Professional or technical or managerial		0.74 (0.42-1.29)		0.80 (0.45-1.39)
Clerical		0.73 (0.38-1.39)		0.79 (0.42-1.49)
Sales		0.69 (0.42-1.13)		0.74 (0.46-1.21)
Agricultural self-employed		0.82 (0.50-1.35)		0.91 (0.56-1.48)
Service		0.68 (0.41-1.14)		0.75 (0.45-1.25)
Skilled manual		0.91 (0.56-1.48)		0.97 (0.60-1.56)
Unskilled manual		0.52 (0.29-0.95)^∗^		0.58 (0.32-1.04)
Religion				
Catholic (ref)				
Protestant		1.04 (0.87-1.24)		1.00 (0.83-1.19)
Other Christians		0.90 (0.69-1.18)		0.86 (0.66-1.12)
Muslim		0.88 (0.70-1.10)		0.96 (0.76-1.21)
Other		0.61 (0.38-0.98)^∗^		0.67 (0.42-1.06)
Media exposure				
No (ref)				
Yes		1.12 (0.92-1.36)		1.08 (0.89-1.31)
Economic status				
Quintiles 1 (ref)				
Quintiles 2		1.51 (1.16-1.98)^∗∗^		1.32 (1.00-1.74)^∗^
Quintiles 3		1.59 (1.18-2.13)^∗∗^		1.39 (1.01-1.90)^∗^
Quintiles 4		1.93 (1.39-2.69)^∗∗∗^		1.69 (1.17-2.44)^∗∗^
Quintiles 5		2.19 (1.51-3.16)^∗∗∗^		1.90 (1.25-2.88)^∗∗^
Decision-making				
No (ref)				
Yes		1.04 (0.89-1.20)		0.98 (0.85-1.14)
Skill ANC				
No (ref)				
Yes		18.25 (10.88-30.62)^∗∗∗^		18.54 (11.05-31.10)^∗∗∗^
Sex of HH head				
Male (ref)				
Female		1.07 (0.88-1.30)		1.03 (0.85-1.26)
Distance to HF				
Big problem (ref)				
Not a big problem			1.28 (1.11-1.48)^∗∗^	1.19 (1.02-1.39)^∗^
Place of residence				
Urban (ref)				
Rural			0.89 (0.70-1.15)	1.08 (0.83-1.41)
Region				
Adamawa (ref)				
Centre (without Yaoun)			1.44 (0.89-2.35)	0.98 (0.59-1.63)
Douala			1.38 (0.82-2.31)	0.91 (0.54-1.55)
East			1.76 (1.14-2.71)^∗^	1.34 (0.85-2.13)
Far-North			1.05 (0.71-1.56)	0.92 (0.61-1.39)
Littoral (without Dou)			1.17 (0.70-1.95)	0.72 (0.43-1.22)
North			0.32 (0.20-0.50)^∗∗∗^	0.27 (0.17-0.43)^∗∗∗^
North-West			2.40 (1.49-3.86)^∗∗∗^	1.44 (0.88-2.36)
West			1.27 (0.78-2.06)	0.77 (0.47-1.27)
South			3.08 (1.89-5.02)^∗∗∗^	2.09 (1.27-3.45)^∗∗^
South-West			1.53 (0.78-3.03)	1.04 (0.52-2.07)
Yaounde			1.23 (0.74-2.05)	0.87 (0.52-1.47)
Community literacy level				
Low (ref)				
Medium			1.22 (0.89-1.67)	0.98 (0.71-1.34)
High			1.36 (0.95-1.96)	1.10 (0.76-1.59)
Community socioeconomic status				
Low (ref)				
Moderate			1.21 (0.93-1.56)	1.08 (0.83-1.40)
High			1.21 (0.88-1.67)	1.05 (0.75-1.47)
Random effect result				
PSU variance (95% CI)	0.77 (0.60-0.98)	0.45 (0.33-0.61)	0.31 (0.21-0.44)	0.26 (0.18-0.40)
ICC	0.19	0.12	0.08	0.07
LR test	308.19	121.02	71.58	52.73
Wald chi-square and p-value	Reference	*χ* ^2^ = 230.37, *P* < 0.001	*χ* ^2^ = 215.02, *P* < 0.001	*χ* ^2^ = 332.48, *P* < 0.001
Model fitness				
Log-likelihood	-2988.28	-2775.28	-2891.00	-2723.31
AIC	5980.56	5616.57	5820.01	5546.63
PSU	427	427	427	427
*N*	5,013	5,013	5,013	5,013

Notes: ^∗^ significant at *P* < 0.05, ^∗∗^ significant at *P* < 0.01, ^∗∗∗^ significant at *P* < 0.001; ref: reference; AIC: Akaike Information Criterion.

## Data Availability

Data for this study were sourced from Demographic and Health surveys (DHS) and available here: https://dhsprogram.com/methodology/survey/survey-display-511.cfm.

## Abbreviations

ANC: Antenatal care
CDHS: Cameroon demographic and health survey
ICC: Intracluster correlation
PC: Preventive chemotherapy
STH: Soil-transmitted helminths.
